# Real-life monocentric experience of venetoclax-based regimens for acute myeloid leukemia

**DOI:** 10.3389/fonc.2023.1149298

**Published:** 2023-03-27

**Authors:** Mariarita Sciumè, Alessandro Bosi, Marta Canzi, Giusy Ceparano, Fabio Serpenti, Pasquale De Roberto, Sonia Fabris, Elena Tagliaferri, Francesca Cavallaro, Francesco Onida, Nicola Stefano Fracchiolla

**Affiliations:** ^1^ Hematology Unit, Fondazione Istituto di Ricovero e Cura a Carattere Scientifico (IRCCS) Ca’ Granda Ospedale Maggiore Policlinico, Milan, Italy; ^2^ Department of Oncology and Hemato-Oncology, Università Degli Studi di Milano, Milan, Italy

**Keywords:** venetoclax, acute myeloid leukemia, azacitidine, decitabine, relapse

## Abstract

**Introduction:**

Combination of venetoclax and hypomethylating agents (HMAs) has become a standard of care in acute myeloid leukemia (AML) aged >75 years or who have comorbidities that preclude intensive induction chemotherapy.

**Methods:**

We conducted a monocentric retrospective analysis on adult patients affected by treatment-naïve AML not eligible for standard induction therapy or refractory/relapsed (R/R) AML treated with venetoclax combinations outside clinical trials. Venetoclax was administered at the dose of 400 mg/daily after a short ramp-up and reduced in case of concomitant CYP3A4 inhibitors.

**Results:**

Sixty consecutive AML were identified. Twenty-three patients (38%) were affected by treatment-naïve AML and 37 (62%) by R/R AML. Median age was 70 years. Among R/R AML 30% had received a prior allogeneic stem cell transplantation (allo-HSCT). In combination with venetoclax, 50 patients (83%) received azacitidine. Antifungal prophylaxis was performed in 33 patients (55%).

Overall response rate was 60%, with 53% of complete remission (CR; 78% for treatment-naïve and 49% for R/R, p 0.017). Median overall survival was 130 days for R/R patients and 269 days for treatment-naïve patients; median event free survival was 145 days for R/R cohort and 199 days for treatment-naïve AML.

Measurable residual disease was negative in 26% of evaluable patients in CR/CR with incomplete hematologic recovery after 2 cycles and in 50% after 4 cycles, with no significant association with survival.

Eleven patients (18%) received an allo-HSCT after venetoclax combinations. Most common grade 3/4 adverse events were infectious (51% of the patients), or hematological without infections (25% of the patients). Use of CYP3A4 inhibitors was associated with a trend to shorter cytopenias and with a lower rate of infections. Invasive fungal infections were less frequent among patients receiving azole prophylaxis (6% vs 26%; p 0.0659).

**Discussion:**

Venetoclax-based regimens are a viable option for AML considered not eligible for standard induction therapy and a valid rescue therapy in the R/R setting.

Azole prophylaxis did not significantly affect response and it was associated with a lower rate of invasive fungal infections. Despite a limited number of patients, the association of venetoclax and HMAs proved to be also a feasible bridging therapy to transplantation.

## 1 Introduction

Acute myeloid leukemia (AML) is the most common acute leukemia in the adult population and largely affects elderly patients, with a median age of 68 years (1). Poor outcomes are described in older patients who are not candidates to intensive chemotherapy because of comorbidities, performance status, and a higher frequency of adverse-risk cytogenetics (2). Historically, such patients were referred to supportive care or treated with non-curative-regimens, such as single-agent hypomethylating agents (HMAs) or low-dose cytarabine (LDAC), with modest response rates (3).

B-cell lymphoma 2 (BCL-2) is an anti-apoptotic protein localized on leukocytes and leukemia stem cells (4). Venetoclax is an oral BCL-2 homology 3 - mimetic, highly selective for BCL-2, initially administered in relapsed/refractory (R/R) AML as single-agent with a modest overall response rate (ORR) of 19% (5). Subsequently, it was tried in combination with azacytidine, decitabine or LDAC (6–9), which lead to its approval for the treatment of newly-diagnosed AML in adults aged ≥75 years, or who have comorbidities that preclude use of intensive induction chemotherapy. In this population venetoclax has become a standard of care with an ORR of about 70%, with notable efficacy even in the adverse genetic risk subgroup (6–9).

In the setting of R/R AML, few promising retrospective data are available with venetoclax single agent or in combination, while the utility of venetoclax in the post-transplant setting remains poorly investigated. Venetoclax-based regimens generally display an acceptable safety profile, with febrile neutropenia, gastrointestinal and hematological adverse events (AEs) as the most commonly reported toxicities (6–9). Real world data for venetoclax-based regimens in AML are accumulating (10–18) and come with the benefit of faithfully representing patients’ outcomes in variable clinical settings, with a lower risk of enrollment bias and sometimes exploring off-label uses.

Moreover, some topics are still debated in the setting of venetoclax-based regimens for AML, such as their ability to induce remission with negative measurable disease, their use as chemotherapy-free bridge to transplantation, and the role of concurrent antifungal azole prophylaxis, which may influence venetoclax pharmacokinetics. The efficacy of venetoclax combinations in transformed AML from pre-leukemic myeloid neoplasms, with or without previous HMA exposure, is also poorly explored to date.

Here we report efficacy and safety data from 60 consecutive AML patient treated at our institution with venetoclax-based regimens, both in the front-line and R/R setting.

## 2 Patients and methods

We conducted a retrospective analysis on adult patients (>18 years old) affected by treatment-naïve AML not eligible for standard induction therapy or R/R AML, who started venetoclax combination regimens between November 2017 and December 2021.

Venetoclax was used in-label for first line therapy or obtained for an off-label use by the Agenzia Italiana del Farmaco (AIFA) “Fondo 5%” for R/R AML.

All diagnoses were performed according to the World Health Organization (WHO) classification (19).

Both AML genetic risk stratification and response criteria were assessed according to 2017 European LeukemiaNet (ELN) consensus (20). *NPM1*, *IDH1/2*, *CEBPA* mutations were detected by Sanger sequencing, while *RUNX1-RUNX1T1*, *CBFB-MYH11*, *FLT3* mutational status was studied by polymerase chain reaction (PCR). *FLT3*-internal tandem duplication (ITD) allele ratio (AR) was calculated by quantitative fragment analysis.

Measurable residual disease (MRD) evaluation was carried out for all patients achieving a complete remission (CR), including patients with incomplete peripheral counts recovery. In the case of *NPM1* mutated patients, MRD evaluation was carried out by quantitative real-time polymerase chain reaction (RT-qPCR) (maximum sensitivity: 10^−6^). In the case of patients lacking a suitable molecular marker, MRD monitoring was carried out by flow cytometry using a leukemia-associated immunophenotypic profile (LAIP)-based approach (21). MRD was analyzed after 2^nd^ and 4^th^ cycle of venetoclax-combinations. This study was conducted in accordance with the Declaration of Helsinki.

### 2.1 Treatment schedules

Venetoclax was administered at a 400 mg daily dose, after a short ramp-up during the first cycle (100, 200, 400 mg per day at day 1, 2 and further, respectively). Most patients were hospitalized during the ramp-up days of cycle 1 and received tumor lysis syndrome (TLS) prophylaxis with allopurinol or febuxostat; the following cycles of therapy were administered in the outpatient setting. Venetoclax dosage was reduced to 50 or 100 mg daily in case of concomitant strong CYP3A4 inhibitors (e.g. posaconazole) and 200 mg daily in case of moderate CYP3A4 inhibitors (e.g. isavuconazole). In case of posaconazole for antifungal prophylaxis, the ramp-up schedule was 10 mg day 1, 20 mg day 2, 50 mg day 3, 100 mg (or 50 mg) day 4 and further.

As for antifungal prophylaxis, by November 2020 our institution introduced a policy of routine azole use in all patients receiving venetoclax combinations since beginning of treatment until reaching of an absolute neutrophil count (ANC) stably >1.0 x 10^9^/L. Before that date, routine antifungal prophylaxis was not offered, in such way that two different subsets of patients – one receiving azole prophylaxis and one without – could be obtained. Azoles were chosen as opposed to echinocandins due to an easier compliance with an oral therapy (azoles) than with daily i.v. therapy (micafungin, the only echinocandin approved in Italy for prophylaxis in hematological patients). Prophylaxis was continued until stable resolution of neutropenia (usually until ANC >1.0x 10^9^/L).

Venetoclax was combined with subcutaneous azacitidine 75 mg/m^2^ for 7 days with a 2-day break or 7 consecutive days, intravenous decitabine 20 mg/m^2^ through days 1-5/28, or cytarabine at different dosage. Venetoclax was administered in a continuous schedule in 28-day cycles for the first cycle.

Our cytopenia management was very similar to those of VIALE-A study (9): in case of incomplete count recovery after the achievement of blast clearance, venetoclax was interrupted from day 29 until ANC ≥0.5 x 10^9^/L or up to 14 days. Next cycle of HMA was also delayed until ANC ≥0.5x10^9^/L or up to 14 days. For subsequent cycles in patients with remission, a new grade 4 neutropenia lasting >1 week required venetoclax interruption after completion of the cycle, and until ANC ≥ 0.5 x 10^9^/L or up to 14 days (unless clinically necessary to interrupt drug within cycle). For subsequent grade 4 neutropenia or thrombocytopenia lasting >1 week, the next treatment cycle was delayed until ANC ≥0.5x10^9^/L or platelet count ≥50x10^9^/L or up to 14 days. In such cases, venetoclax would then be administered for 21/28 days. In case of new severe cytopenia onset, further venetoclax reduction steps were 10/28 and 7/28 days. The last step of reduction was azacitidine dose adjustment at 50% of the initial dosage.

A bone marrow (BM) aspirate was performed to rule out disease persistence before any dose reduction.

Patients usually received additional prophylaxis with acyclovir and trimethoprim/sulfamethoxazole. Filgrastim support was administered in case of delayed neutrophil recovery and BM CR. Antiemetic prophylaxis was given as per local practice only during the HMA administration. Other supportive measures included red blood cell and platelet transfusions as clinically indicated.

### 2.2 Statistical analysis

Response rates were calculated with all patients at the denominator, including those who died before bone marrow evaluation. Event free survival (EFS) was defined as the time from VEN initiation to documentation of refractoriness, relapse or death from any cause. Overall survival (OS) was calculated since time of starting venetoclax to time of death from any cause, or censored at last follow-up. EFS and OS were all assessed using the Kaplan-Meier method and compared between groups using the log-rank test; p values <0.05 were considered significant. A two-tailed Fisher’s exact test was used for dichotomic variables. All statistical analyses were performed using R software version 4.2.1.

## 3 Results

A total of 60 consecutive AML patient treated with venetoclax-based combinations were considered in this study. Twenty-three patients (38%) had treatment-naïve AML, while 37 patients (62%) had R/R AML. As for dosing schedules, 22/60 (37%) patients received 400 mg/d without interacting drugs, 3/6 (5%) received dose reductions due to toxicity, 10/60 (17%) received 200 mg/day due to concomitant moderate CYP3A4 inhibitors, 6/60 (10%) received 100 mg due to concomitant isavuconazole or posaconazole, and 19/60 (32%) received 50 mg due to concomitant posaconazole (n = 18) or voriconazole (n = 1). The main characteristics of the patients are summarized in **Table 1**.

**Table 1:** Patients’ characteristics: for continuous variables, median values (range) are reported.

FEATURE	Total (n=60)	Treatment-naive AML (n= 23)	Relapsed/Refractory AML (n=37)
Age and sex			
Median age (range), years	70 (30 - 84)	75 (55 – 84)	65 (30-79)
> 65 years old – no. (%)	40 (67)	21 (91)	19 (51)
Male – no. (%)	35 (58)	14 (61)	21 (57)
ELN 2017 Risk – no. (%)			
Adverse	21 (35)	9 (39)	12 (32)
Intermediate	32 (53)	13 (57)	19 (51)
Favorable	7 (12)	1 (4)	6 (16)
Molecular mutations – no. (%)			
*RUNX1-RUNX1T1*	0	0	0
*CBFB-MYH11*	0	0	0
*FLT3 ITD*	7 (12)	1 (4)	6 (16)
*FLT3 TKD*	1 (2)	0 (0)	1 (3)
*NPM1*	10 (17)	2 (9)	8 (22)
*MLL* rearrangement	0	0	0
*CEBPA* biallelic	1 (2)	0	1 (3)
*IDH1*	2 (3)	2 (9)	0
*IDH2*	4 (7)	2 (9)	2 (5)
Previous hematological disease – no. (%)	20 (33)	11 (48)	9 (24)
MDS	11 (55)	8 (73)	3 (33)
MPN	3 (15)	0	3 (33)
MDS/MPN	6 (30)	3 (27)	3 (33)
Associated drug– no. (%)			
Azacitidine	50 (83)	22 (96)	28 (76)
Decitabine	5 (8)	1 (4)	4 (11)
Cytarabine	2 (3)	0	2 (5)
No associated drug	3 (5)	0	3 (8)
Relapsed status – no. (%)			
First line	23 (38)	23 (100)	0
Salvage 1	14 (23)	0	14 (38)
Salvage ≥2	23 (38)	0	23 (62)
Previous treatment lines for AML			
Intensive chemotherapy	22 (37)	0	22 (59)
Intensive chemotherapy and HMAs	10 (17)	0	10 (27)
HMA monotherapy	5 (8)	0	5 (14)
Previous exposure to HMAs (for AML or pre-AML) – no. (%)			
Yes	22 (37)	5 (22)	17 (46)
No	38 (63)	18 (78)	20 (53)
Prior allogeneic HSCT – no. (%)			
Yes	11 (18)	0	11(30)
No	49 (82)	23 (100)	26 (70)
Allogeneic HSCT after venetoclax – no. (%)			
Yes	11 (18)	4 (17)	7 (19)
No	49 (82)	19 (83)	30 (81)
Venetoclax dose reduction due to CYP3A4 inhibitors – no. (%)			
Yes	35 (58)	16 (70)	19 (51)
No	25 (42)	7 (30)	18 (49)

For categorical variables, numbers (percentage) are reported. AML, Acute Myeloid Leukemia; ELN, European Leukemia Network; HMAs, Hypomethylating Agents; HSCT, Hematopoietic Stem Cell Transplantation; MDS, Myelodysplastic Syndromes; MPN, Myeloproliferative Neoplasms.

### 3.1 Treatment naïve AML

Median age was 75 years (range 55 - 84), with 21 of them (91%) being older than 65 years. Fourteen (61%) were male. Thirteen (57%) had an intermediate-risk disease, 9 (39%) had an adverse risk and only one (4%) a favorable risk. We found *FLT3-ITD* mutation in 1 patient, while mutations in *NPM1*, *IDH1* and *IDH2* were found in 2 patients each. Notably, 11 (48%) had a transformed AML from a myelodysplastic syndrome (MDS), myeloproliferative neoplasm (MPN) or MDS/MPN overlap.

Consequently, 5 patients (22%) had already been exposed to HMAs to treat the pre-leukemic conditions. Venetoclax was associated with azacitidine in 22 cases (96%) and decitabine in only one patient (4%). Of note, CR was achieved in 8 patients (35%), with a composite CR [CR/CR with incomplete hematologic recovery (CRi)] rate of 52%. BM blast clearance (CR/CRi/Morphologic leukemia-free state, MLFS) was observed in 17 (74%) patients, while 1 case (4%) had partial remission. Five patients (22%) did not respond or died before evaluation.

**Figure 1:**
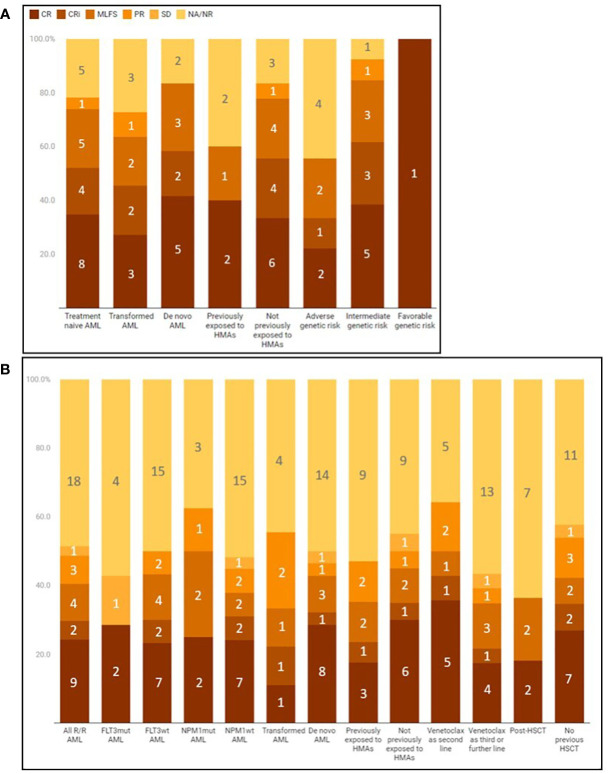
Best response to venetoclax combinations in different subgroups of treatment-naïve **(A)** or relapsed/refractory **(B)** AML. Responders can be visualized as a percentage of the subgroup (vertical axis) or as raw counts (numbers inside the bar graph boxes). CR, complete remission; CRI, complete remission with incomplete hematological recovery; HMAS, hypomethylating agents; MLFS, Morphological Leukemia Free State; NA, not available due to patient's death before evaluation; NR, non-responder, PR, partial response; SD, stable disease; VEN, venetoclax.

Response data according to clinical and biological characteristics were summarized in **Figure 1A**.

When restricting to transformed AML, a CR/CRi rate of 45% and a BM blast clearance rate of 64% were observed. Response rate was considerable even in patients previously exposed to HMAs for pre-leukemic conditions (BM blast clearance in 3/5 patients, 60%). BM blast clearance appeared to occur more frequently in favorable/intermediate risk patients than in adverse-risk, although at no statistical significance (n = 12/14, 86%, vs 5/9, 56% respectively; p 0.16).

Median time to best response was 1 month (1 – 12 months), with 10 patients (59%) achieving it after the first cycle, 2 (12%) after 2 cycles, and 5 (29%) after >2 cycles. Four patients received an allogeneic stem cell transplantation (HSCT), aged 60, 65, 74 and 73 years. Choice of the association of venetoclax and HMAs as induction therapy in such patients was guided by age and clinical conditions at the time of AML diagnosis. During the follow-up the improvement of clinical conditions and the achievement of CR allowed the patients to be candidates for transplantation, with an age-adapted conditioning.

Concerning venetoclax dose, 7 (30%) patients received venetoclax without concomitant interactions, while the others received a reduced dose. A trend to a higher response was associated with venetoclax full-dose compared to reduced dose: blast clearance 7/7 (100%) vs 10/16 (62.5%), respectively, p 0.12.

Median OS was 269 days (range 26-500), whereas median EFS was 199 days (range 26-469). ELN adverse genetic risk was associated with a worse OS (median 95 days, range 26-500) as compared with favorable/intermediate genetic risk (median 299 days, range 20-508).

For patients with secondary AML, median OS was 206 days (range 28-416) and median EFS was 175 days (range 53-416). *De novo* AML had median OS of 300 days (range 26-508) and median EFS of 287 days (range 26-469).

### 3.2 Relapsed/refractory AML

Median age was 65 years (range 30 - 79), with 19 (51%) being >65 years old. Half had an ELN intermediate risk (n =19, 51%), 12 (32%) had adverse-risk and only 6 (16%) a favorable-risk disease.

Mutational analysis at first diagnosis revealed 6 patients (16%) with *FLT3-ITD* mutation, 1 (3%) with *FLT3-TKD* mutation, 8 (22%) with *NPM1* mutations, 1 (3%) with *CEBPA* biallelic mutation and 2 (5%) with *IDH2* mutations. Nine (22%) patients had a secondary AML transformed from MDS, MPN or MDS/MPN.

Concerning previous treatment lines for AML, 22 (59%) patients had received intensive chemotherapy +/- *FLT3* inhibitors, 5 (14%) had received only HMAs and 10 (27%) had received both intensive chemotherapy and HMAs. Notably, 17 (46%) patients had been exposed to HMAs before venetoclax, either to treat AML or pre-leukemic conditions. Overall, 14 (38%) received venetoclax- based regimens as second-line for AML, whereas 23 (62%) were receiving it as a third or further line.

Eleven (30%) patients had prior history of allogeneic HSCT.

Venetoclax was associated with azacitidine in 28 (79%) patients, decitabine in 4 (11%), cytarabine in 2 (5%) and given alone in 3 (8%) cases.

CR was achieved in 9 (24%) patients, with a composite CR/CRi of 29% (n =11) and a CR/CRi/MLFS rate of 41% (n = 15). Three patients achieved PR as best response, accounting for an ORR of 49% (n = 18). Eighteen patients (49%) did not respond or died before evaluation of response, whereas one patient obtained a stable disease.

Response data according to clinical and biological characteristics were summarized in **Figure 1B**.

Median time to best response was 1.4 months (range 0.5 – 3.9), with 10 patients (56% of responders) achieving it after the first cycle. Seven R/R AML patients received allogeneic HSCT after venetoclax/HMAs (median age 57 years, range 48 – 74).

Eventually, in our R/R cohort, estimated median OS and EFS were 130 days and 145 days, respectively.

ELN adverse genetic risk was associated with a median OS of 88 days (range 9-648), while favorable/intermediate genetic risk patients had a median OS of 178 days (range 12-604).

### 3.3 MRD evaluation

Of the 23/60 patients in CR/CRi (12/23 with treatment-naïve AML and 11/37 R/R AML), 3 had a *NPM1-*mutated AML (1 treatment-naïve and 2 R/R). Sixteen patients underwent MRD evaluation by flow cytometry, while 4 patients were not evaluable due to absence of LAIP or to unavailability of suitable BM samples. Five of 19 evaluable patients (26%) were MRD-negative after 2 cycles of venetoclax-HMAs (4 with intermediate-risk and 1 with adverse-risk disease). Two MRD-negative patients relapsed with an EFS of 6.0 and 11.1 months, one died in remission after 8.1 months and two were alive in CR at 11.6 and 25.0 months (the latter received consolidation with allogeneic HSCT). When considering the 4^th^ cycle as landmark for MRD, 12 patients were evaluable (after excluding patients who experienced relapse, death or allogeneic HSCT earlier). Six of 12 patients (50%) were MRD-negative. Of those, two experienced relapses after 10.9 and 6.0 months, while four were alive after a median follow up of 8.8 months. EFS was not significantly different between MRD-positive and -negative patients both after 2 and after 4 cycles of venetoclax-combinations (p 0.66 and 0.96, respectively). One of the 3 *NPM1*-positive patients needed venetoclax discontinuation due to diagnosis of decompensated liver cirrhosis after the fifth cycle of venectoclax-azacitidine, continuing with HMA only. Notably, her *NPM1* negativity on BM and peripheral blood is still confirmed after 11.4 months from venetoclax withdrawal.

### 3.4 Safety profile

In the whole cohort analysis, 48 patients (80%) developed grade 3 or 4 complications, which were solely hematological in 15 patients (25%) and infectious in 31 patients (52%).

One event of severe TLS was reported, leading to severe acute kidney failure and need for hemodialysis; in such patient, renal impairment slowly improved until abrogation of dialysis need. One patient discontinued venetoclax due to severe liver impairment and cirrhosis, even if the cause remains unclear.

There were 47 hospitalizations or prolonged hospital stays due to infections, in 29/60 (48%) patients, with a median duration of hospitalization of 22.3 days (range 4 – 77). Most common grade 3/4 infections were febrile neutropenia in 9 patients (15%), bacteriemia in 12 (20%), of which 8 developed septic shock, and invasive fungal infections (IFIs) in 10/60 patients (17%). Of the latter, there were 8 fungal pneumonias, 1 complicated urinary candidosis and 1 systemic candidosis.

All patients who developed IFIs had never received, or had interrupted, antifungal prophylaxis, except one patient who developed systemic candidosis while on isavuconazole. One patient received diagnosis of AML evolution and aspergillosis at the same time and was by definition ineligible for prophylaxis. Excluding the latter, IFIs were more frequent among patients who had never received fungal prophylaxis, 7/27 (26%), vs those who received it at least until neutrophil recovery 2/32 (6%), p 0.0659.

Median duration of neutropenia and thrombocytopenia along the first venetoclax cycle in patients achieving MLFS/CRi/CR were 23.5 and 18 days, respectively. Duration of neutropenia trended to lower values among patients receiving CYP3A4–inhibitors than among patients who did not (median 34.5 days vs 20.5, respectively; p 0.19), as well as duration of thrombocytopenia (median 17 days vs 21.5 days, respectively; p 0.923). Patients who experienced grade 3/4 infections were fewer in the CYP3A4-interactions group than in the no-interaction group (n = 16/35, 46%, vs 14/25, 56%, respectively; 0.2936).

Eleven patients (18%) needed reduction of venetoclax exposure window: 21 days for 4 (7%) patients, 14 days for 2 (3%) patients, 10 days for 2 (3%) patients and 7 days for 3 (5%) patients. Reduction of HMA to 50% of the total dose was applied to 2 (3%) patients.

In the context of post-transplant venetoclax treatment, 9 (82%) patients experienced grade ≥3 toxicity (8 hematological, 3 infectious, 1 gastroenteric). Five (45%) patients needed to prematurely interrupt venetoclax after a median time of 35 days (range 25-46).

## 4 Discussion

We report real-world data of 60 AML patients treated with venetoclax-based regimens either in first line or in R/R setting.

In the first-line context, we observed a composite CR rate of 52%, consistent with the phase III study and with other real world reports. No statistically significant differences in rate of blast clearance (CR/CRi/MLFS) were noted in subgroup analysis. Of interest, considerable response rates were observed in patients with secondary AML, where we observed a CR/CRi rate of 45% and a CR/CRi/MLFS rate of 64%. These data are very similar to induction response obtained through intensive chemotherapy with CPX-351 in real-world experience (22), with the associated benefits of a less intensive approach. Notably, previous exposure to HMAs was not apparently associated with a worse response in our analysis (CR/CRi/MLFS rate of 3/5, 60%), suggesting that venetoclax + HMAs could be effective even in high-risk patients with AML evolving from azacitidine-treated MDS. Median OS was 8.8 months, slightly lower than other reports (10, 11, 19).

In the R/R context, our data provide further evidence that some disease remission can be induced by venetoclax-based regimens even in heavily pre-treated patients (62% had already received at least 2 treatment lines). Particularly, the observed CR/CRi rate in our 37 patients was 30% (11/37), consistent with other published series (12–15, 17, 18).

As previously observed in the context of R/R AML (12), previous exposure to HMA for AML treatment slightly trended to an inferior rate of CR/CRi/MLFS. However, in our report the rate of CR/CRi/MLFS in these patients (n = 17) was not negligible (35%).

Furthermore, a promising composite CR rate was observed in patients who relapsed after allogeneic HSCT (4/11, 36%), a setting where few alternatives are usually available.

Median OS in our R/R AML cases was 4.3 months, while the range of other case series reported in literature is between 5 and 8.1 months (12, 13, 17, 18).

In spite of a low number of patients, our results suggest that venetoclax-based regimens might successfully be used as a bridge to allogeneic HSCT and demand wider and prospective studies.

We report a rate of MRD negativity after 2^nd^ and 4^th^ cycle of 26% and 50% of evaluable patients, respectively. Such rates are lower than the ones reported by Maiti et al. (23), possibly because our cohort included a large proportion of heavily pretreated patients and a low proportion of favorable-risk patients.

Venetoclax regimens were associated with grade 3/4 adverse events in 48 (80%) patients, with 15 (25%) having purely hematological events, and 31 (52%) having grade 3/4infections. The only event of severe lysis syndrome was reversible. Such safety profile recapitulates the one observed in clinical trials (6, 7, 9).

Our data suggest that antifungal prophylaxis needs to be offered during venetoclax regimens, as we observed a rate of grade 3/4 invasive fungal infections in 26% of patients who did not receive azoles. In this respect, azole prophylaxis during induction (i.e. until blast clearance and neutrophil recovery with ANC >1x 10^9^/L) was associated with a reduced rate of fungal infections, at borderline statistical significance. Such policy may represent an acceptable alternative to a logistically challenging echinocandin prophylaxis. Notably, concomitant azole prophylaxis did not seem to affect response rate, nor to extend cytopenias, provided that an appropriate dose reduction of venetoclax was performed, as per clinical standard. The trend to a lower extension of cytopenias in patients receiving CYP3A4 inhibitors is discordant from literature (24), whereby it is speculated that interactors prolong exposure to venetoclax and, consequently, increase hematological toxicity. We found two possible explanations for our contrasting finding: on one side, use of CYP3A4 inhibitors roughly coincided with a time when experience with venetoclax had consolidated worldwide and at our center as well, such that we began to program more temporary interruptions of venetoclax or reductions of exposure windows. On the other side, most dose adjustments consisted in reducing venetoclax to 50 mg when concomitant posaconazole was administered. Such dose is lower than the one used in literature, whereby 70 to 100 mg were usually considered. Patients who experienced grade 3/4 infectious complications were fewer among the CYP3A4-interactions group, accordingly with shorter neutropenia and with prevention of fungal infections. Eventually, we remark that 18% of our patients needed a reduction of venetoclax exposure, occasionally together with azacitidine reductions. We believe that such reductions can be safely performed after assessing bone-marrow blast clearance (BM blasts <5%) in case of persisting cytopenias. In particular, our cytopenia management was very similar to those of VIALE-A study (9) as explained in the method section.

In conclusion, the results of this real-life monocentric study confirmed that venetoclax-based regimens are a viable and safe option as first-line therapy for AML unfit patients and a valid rescue therapy in the R/R setting. Furthermore, although based on a small subgroup of patients, the association of venetoclax and HMAs proved to be also a feasible bridging therapy to transplantation.

## Data availability statement

The original contributions presented in the study are included in the article, further inquiries can be directed to the corresponding author.

## Ethics statement

The studies involving human participants were reviewed and approved by Comitato Etico Milano Area B. The patients/participants provided their written informed consent to participate in this study.

## Author contributions

Conceptualization: MS, AB, and NF. Data curation: MS, AB, MC, GC, FS, PD, SF, ET, FC, and NF. Writing – original draft preparation: MS, AB, and NF. Writing – review & editing: MS, AB, and NF. Visualization: MS, AB, MC, GC, FS, PD, SF, ET, FC, and NF. Supervision: MS, AB, FO, and NF. All authors approved the submitted version. All authors contributed to the article and approved the submitted version.
